# Syphilis, Notes on

**Published:** 1874-04

**Authors:** 


*THE MEDICAL EXAMINER—January*, 1874:

NOTES ON SYPHILIS.—Lancereaux on the treatment of acquired syphilis (*La France Medicale*, Nov. 5, 1873:) 1. Period of primary accident, or syphilitic chancre: No mercury should be employed; the regime should be slightly tonic, ferruginous, and hydro-therapeutic. Mercury is to be exhibited only in order to procure resolution of indurations which are indolent in disappearing, and is the more useful and necessary as the ganglionic system is more profoundly affected. In all other cases observe scrupulously cleanliness, and employ lotions of aromatic wine, or alcohol mixed with a variable quantity of water; and dress simply with calomel ointment, or dry lint. Cauterization is useless, if not dangerous.

2. Period of secondary accidents: When local manifestations, though imminent, are not yet declared, and the patient is in a prodromic stage, we find intense cephalalgia, general lassitude, wandering pains, and moral depression. These point to a speedy explosion of the disease. Mercury is not yet indicated, but laxatives, if the secretions are disturbed; iron, if there be chloro-anæmia; and, as a rule, rest, by the aid of opium and baths. Mercury is to be exhibited when exanthemata occur, even if these be of the roseolous and papular varieties, for which M. Diday considers such a medication valueless. Discontinue the mineral on subsidence of the eruption, lest anæmia or *obesity* (sic?) be induced. The author does not recommend the administration of mercury by inunction, or subcutaneous injection, but prefers the use of the bichloride or protochloride internally, especially the former, and discontinues the remedy on the disappearance of all syphilitic manifestations. The intercurrent indications are to be met. Baths, and the iodide of potassium, are useful in case of syphilitic fever, and at the outset of secondary manifestations.

3. Period of tertiary accidents: Iodide of potassium is generally indicated. In cases of visceral complication, calomel, in minute doses, should be tried. In some intestinal derangements, due to lesion of the hæmatopœtic glands (liver and amyloid spleen), mercurial friction is recommended, and the use of nitric lemonade (Bud.) Ulcers of foul aspect require glycerine, alcohol, tincture of iodine, sulphate of copper, nitric acid, perchloride of iron, corrosive sublimate, iodoform, more or less concentrated solution of chloral, or meta-chloral (Duj.-Beaumetz.) Syphilitic arthropathy, osteocopic pain, and osteoperiostitis, require vesication. For rebellious exostoses apply vesicatories, and dress with tincture of iodine or neapolitan ointment and emollient cataplasms.

